# Estimating the cost consequence of the early use of botulinum toxin in post-stroke spasticity: Secondary analysis of a randomised controlled trial

**DOI:** 10.1177/02692155221133522

**Published:** 2022-11-03

**Authors:** Cameron Lindsay, Ioan Humphreys, Ceri Phillips, Anand Pandyan

**Affiliations:** 1 46 Bangor Road, Holywood, Down, UK; 2 Swansea University, First Floor, Vivian Building, Singleton Campus, Swansea, UK; 3 Bournemouth University, Bournemouth Gateway Building (Rm 507), St Pauls Lane, Bournemouth, UK

**Keywords:** Stroke, spasticity, botulinum toxin, economic evaluation

## Abstract

**Objective:**

To estimate the cost-consequence of treating spasticity early with botulinum toxin in the acute stroke unit.

**Design:**

Secondary cost-consequence analysis, using data from a double-blind randomised-controlled trial.

**Setting:**

Single-centre specialised stroke unit.

**Subjects and Interventions:**

Patients with Action Research Arm Test grasp-score of <2 and who developed spasticity within six weeks of a first stroke were randomised to receive injections of: 0.9% sodium-chloride solution (placebo) or onabotulinumtoxin-A (treatment).

**Main measures:**

Resource use costs were calculated for the study. Mean contracture costs for each group were calculated. The Barthel Index and Action Research Arm Test were used to generate a cost per unit of improvement.

**Results:**

There were no significant differences associated with early treatment use. The mean contracture cost for the treatment group was £817 and for the control group was £2298 (mean difference = −£1481.1(95% CI −£2893.5, −£68.7) (*p* = 0.04). The cost per unit of improvement for the Barthel Index was −£1240 indicating that the intervention costs less and is more effective. The cost per unit of improvement for the Action Research Arm Test was −£450 indicating that the intervention costs less and is more effective.

**Conclusions:**

Treating spasticity early in stroke patients at risk of contractures with botulinum toxin leads to a significant reduction in contracture costs. The cost per improvement of Barthel and Action Research Arm Test indicates that the intervention costs less and is more effective.

**Trial Registration data:**

EudraCT(2010-021257-39) and ClinicalTrials.gov-Identifier:NCT01882556.

## Introduction

The cost of managing stroke places a significant burden on the individual, families and the economy.^1^ These costs significantly increase in stroke patients who develop complications such as spasticity, contractures, pain and pressure sores.^2, 3^ There is a growing body of evidence that demonstrates that spasticity occurs early following stroke.^4^ In patients who do not recover useful arm function, this is likely to lead to contractures, pressure sores and pain.^4^

Botulinum toxin is one treatment that can reduce spasticity^5^ but it is not routinely offered to acute stroke patients. A possible reason for this could be the perception that this drug is expensive.^6^ Apart from one cost-effectiveness study from the results of a randomised-controlled trial,^6^ economic studies have either used retrospective cohort analysis^2^ or economic modelling using expert opinion.^7, 8^

We have previously demonstrated that screening and treating spasticity early, on first presentation after the first stroke in patients who have no useful arm function, with botulinum toxin prevents contractures, reduces pain, and does not interfere with functional recovery (EUBoSS Trial).^5^ This current paper uses results from this randomised-controlled trial to investigate the cost-consequences of using botulinum toxin early in the management of spasticity following stroke.

Using data from the previous trial we aim to

1. Identify the major cost drivers in the management of patients with post-stroke spasticity and contractures.
2. Conduct a cost-consequences analysis of botulinum toxin in the management of patients with post-stroke spasticity and contractures.

## Methods

This study reports on the cost-consequences of the early use of botulinum toxin in post-stroke spasticity. The trial was approved by North West – Greater Manchester South Ethics Committee Reference number 10/H1003/111. It was registered with EudraCT (2010-021257-39) and at ClinicalTrials.gov-Identifier: NCT01882556.

The protocol^9^ and the results related to effectiveness have previously been published.^5^ In brief, in this double-blind placebo-controlled study, patients with an Action Research Arm Test grasp-score ⩽2 and who developed spasticity within six weeks of a first stroke were randomised to receive injections of either 0.9% sodium-chloride solution (placebo) or onabotulinumtoxin-A (treatment). In addition to a range of measures of impairment and hand function, the Barthel Index, and Action Research Arm Test were measured at six months post-stroke.^9^

Further, details regarding participants’ use of health services were documented at two, four, six and 12 weeks following treatment and at six months post-stroke. These included GP visits, hospital visits and admissions. Current medication use and any changes from discharge were documented. Treatments to manage contractures were also recorded. Patients were encouraged to document such activities in a diary and this additional informal questioning was completed at each review to ensure all information was provided.

Resource use costs associated with the study were summarised into relevant categories and valued in £ sterling using a price year of 2017/18. The costs were determined from national published sources of unit costs British National Formulary,^10^ National Health Service reference costs (NHS 2017/2018) and estimated contracture costs.^11^

The Barthel Index and Action Research Arm Test were used to generate a cost per unit of improvement in each of the measures. A one-way sensitivity analysis was undertaken (using IBM SPSS 27) to assess the extent of potential changes in the main cost parameters and outcomes of the treatment using the mean difference and lower and upper bounds of the confidence intervals. The 95% confidence interval of net cost and changes in outcomes were used to generate a series of potential scenarios and explore the changes in the estimated cost per unit of improvement.

## Results

**Figure 1.**
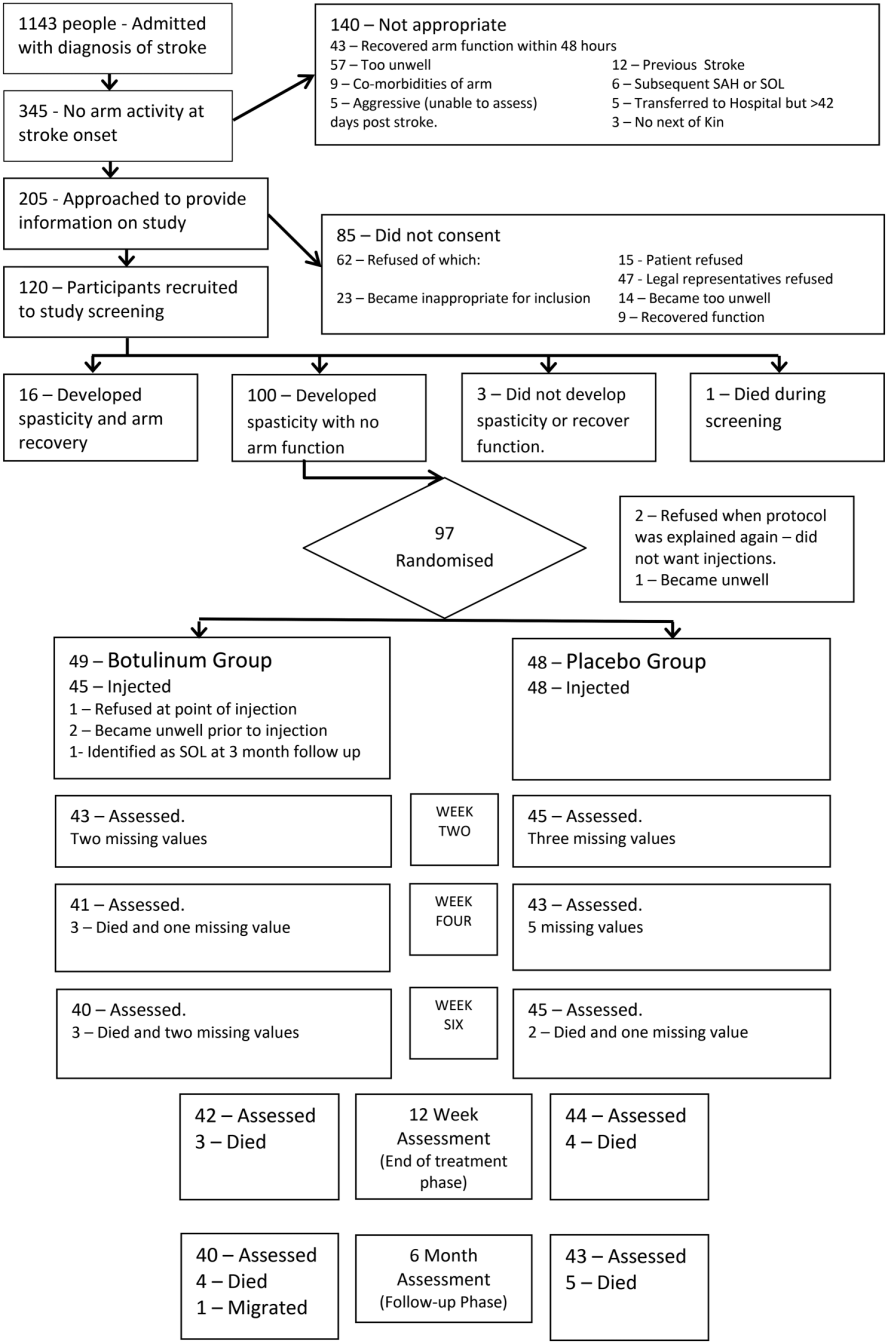
Consort flow chart summarising the flow of participants through the study. The last value was carried forward in cases of missing values. SAH: subarachnoid haemorrhage; SOL: space occupying lesion.

Between January 2012 and December 2013, 93 participants were randomised and received injections (see Figure 1: CONSORT diagram). The treatment group (*n* = 45) and control group (*n* = 48) were similar at baseline.^5^

### Intervention cost

The costs related to the intervention arm of the trial were summed to give the total cost per participant (NHS costs). Where possible, unit costs for the UK were applied (e.g. Personal Social Services Research Unit, British National Formulary^10^) to increase generalisability. These are reported in Table 1.

**Table 1.** Summary of all intervention costs.

Cost items	Unit cost	Total cost	Unit cost source/description	Comments
Therapist (band 6)	£45	£2025	PSSRU (2019) band 6 – Page 121	Based on (*n* = 45)
Drug cost	£250	£11,250	Drug prices vary between trusts (depending on local negotiations), so we are using a mid-estimate of £250	The total dose was 160 units divided between 6 muscles. Since the vials can only be used for one patient and they only come in 200 unit vials the lowest actual cost is an estimated £250. Based on (*n* = 45)
		£13,275	Cost per intervention participant	£295

Resource use analysis is presented in Table 2. There were no significant differences when the costs associated with all prescribed drugs were assessed between groups at discharge, three months and six months. The calculated costs associated with the initial admission and subsequent admissions for both groups were analysed, and no statistically significant difference was identified. The mean total costs associated with hospitalisations were £21,577 in the treatment group and £21,389 in the control group (mean difference £187.6; 95% CI −£3989.3, £4364.5). When all costs were combined, no statistically significant differences were identified (Table 2).

**Table 2.** Between-group analysis of resources and costs.

Measure	Intervention	*N*	Mean	Standard deviation	Mean difference and 95% confidence interval of the difference	*p*-value
Drug cost 3 months (£)	Treatment	41	320	365.8	−20.7 (−251.67, 210.4)	0.859
	Control	45	341	656.6		
Drug cost 6 months (£)	Treatment	40	333	396.1	−23.6 (−267.3, 220.0)	0.847
	Control	43	356	673.6		
Drug cost discharge from hospital (£)	Treatment	42	342	363.2	−23.0 (−237.6, 191.5)	0.831
	Control	46	365	607.1		
Total drug costs (£)	Treatment	42	971	1094.9	−60.1 (−721.8, 601.6)	0.857
	Control	46	1032	1885.8		
Length of stay (days)	Treatment	45	59.3	33.4	−1.4 (−14.2, 11.4)	0.828
	Control	48	60.7	28.6		
Cost of stay (£)	Treatment	45	19,897	9833.8	321.4 (−3477.9, 4120.6)	0.867
	Control	48	20,219	8601.0		
Readmitted length of stay (days)	Treatment	13	15.6	18.2	4.3 (−9.5, 18.2)	0.522
	Control	11	11.3	13.6		
Readmitted cost of stay (£)	Treatment	45	1679	4223.9	508.9 (−1056.6, 2074.5)	0.501
	Control	48	1170	3351.3		
Combined length of stay (days)	Treatment	45	63.8	36.4	0.5 (−13.1, 14.1)	0.939
	Control	48	63.3	29.5		
Overall length of stay costs (£)	Treatment	45	21,577	11,075.2	187.6 (−3989.3, 4364.5)	0.929
	Control	48	21,389	9165.5		
Intervention costs (£)	Treatment	45	295	0.0	-	-
	Control	48	0	0.0		
Contracture costs (£)	Treatment	45	817	2645.7	−1481.1 (−2893.5, −68.7)	0.040
	Control	48	2298	4022.8		
Overall costs (£)	Treatment	45	23,595	11,539.7	−1080.5 (--5867.2, 3706.3)	0.655
	Control	48	24,676	11,682.4		

The mean contracture cost for the treatment group was £817 while the costs for the control group was £2298. This was statistically significant (*p* = 0.04) with a mean difference of −£1481.1 (95% CI −£2893.5, −£68.7).

The change in Barthel Index scores between baseline and six months was lower in the control group (mean 7.3 (SD 6.0)) compared to the treatment group (mean 8.1 (SD 5.0)). The mean difference was 0.8 and not statistically significant (95% CI −1.5, 3.3, *p* = 0.47).

### Sensitivity Analysis

A one-way sensitivity analysis was undertaken to assess the extent of potential changes in the main cost parameters and outcomes of the treatment using the mean difference, lower and upper bounds of the confidence intervals for the Barthel score (Table 3) and the Action Research Arm Test (Table 4).

**Table 3.** Incremental cost of intervention using the Barthel score.

Parameter	Incremental cost of intervention	Incremental Barthel score	Reduction in cost/increase in cost per unit of improvement
Baseline	−£1080.5 (−£5867.2, £3706.3)	0.87 (−1.55, 3.29)	−£1240
Upper 95% bound of net cost	£3706	3.297	£1124
Upper 95% bound of net utility			
Upper 95% bound of net cost	£3706	−1.555	−£2383
Lower 5% bound of net utility			
Lower 5% bound of net cost	−£5867	−1.555	£3773
Lower 5% bound of net utility			
Lower 5% bound of net cost	−£5867	3.297	−£1780
Upper 95% bound of net utility			

**Table 4.** Incremental cost of intervention using the Action Research Arm Test (ARAT) score.

Parameter	Incremental cost of intervention	Incremental ARAT score	Reduction in cost/increase in cost per unit of improvement
Baseline	−£1080.5 (−£5867.2, £3706.3)	2.4 (−6.00, 10.71)	−£450
Upper 95% bound of net cost	£3706	10.71	£346
Upper 95% bound of net utility			
Upper 95% bound of net cost	£3706	−6.00	−£618
Lower 5% bound of net utility			
Lower 5% bound of net cost	−£5867	−6.00	£978
Lower 5% bound of net utility			
Lower 5% bound of net cost	−£5867	10.71	−£548
Upper 95% bound of net utility			

When the base case results of the costs of the intervention are used with the base case results of the Barthel scores, the cost per unit of improvement was −£1240 indicating that the intervention costs less and is more effective.

When the lower 5% bound of net cost (−£5867) is used with the upper 95% bound of net utility (3.297), then the intervention is again seen as costing less and more effective (−£1780) (see Table 3). When the base case results of the costs of the intervention are used with the base case results of the Action Research Arm Test scores, the cost per unit of improvement was −£450 indicating that the intervention costs less and is more effective.

When the lower 5% bound of net cost (−£5867) is used with the upper 95% bound of net utility (10.71), then the intervention is again seen as costing less and more effective (−£548) (see Table 4).

## Discussion

We have previously demonstrated that the early use of botulinum toxin reduced spasticity and contractures after stroke and that the effects lasted for approximately 12 weeks.^9^ In this study, we have demonstrated that the additional treatment with botulinum toxin does not lead to an increase in cost associated with managing these patients.

This indicates that the early use of botulinum toxin for post-stroke spasticity can be cost-saving to the NHS when assessed in terms of gain in the activity of daily living (Barthel Index) and arm function (total Action Research Arm Test). This result contrasts with previously reported cost-effectiveness data that suggested that the base case incremental cost-effectiveness ratio for botulinum toxin type A plus therapy was £93,500.^6^ However, it is possible that the magnitude of difference can be explained by the differences in the two studies:

- The previous study measured response to treatment using the Modified Ashworth Score (an invalidated measure of spasticity),^12^ which has previously been shown to underestimate treatment effects.^13^
- The previous study treatment was initiated in patients who are likely to have established spasticity and/or contractures (mean time to treatment 46 weeks post-stroke).^6^ This contrasts with the current study where treatment was initiated in patients who presented with spasticity prior to contractures being established (mean time to treatment 14 days post-stroke).^5^
- The previous study primarily focused on improving function using a combination of therapy and botulinum toxin^6^ and in the current study,^5^ the primary focus was to prevent contractures.
- In the current study, all patients had an Action Research Arm Test of 0 to 2 at injection,^5^ whereas in the previous study 45% of the participants had an Action Research Arm Test >3.^6^

There are two main limitations in this study. The first is that the study had a small sample size and was not powered to assess cost-effectiveness. As a result, some data was not recorded and is therefore absent from the analysis. For example, the number of therapist contacts following discharge to the community was not recorded. A further limitation is that this study has only been able to assess the health costs rather than additional social costs. While the study recorded details about the number of paid social care services participants received, unpaid family care was not recorded. Secondly, the patients were not monitored longer term, so we have had to estimate the costs associated with treating contractures. Currently, evidence suggests contractures are likely to be common^6^ and are often not managed well. The costs for long-term management of contractures can be high. It may involve treating repeated infections with antibiotics, the treatment of pressure sores and surgery for debridement or to reduce discomfort and allow for basic hygiene. For the purposes of this study, we have used a mean cost for the long-term management of contractures of £9193.^11^ We feel that this is a realistic cost that includes the full direct and indirect costs of contracture management.

This study is the first randomised-controlled study using botulinum toxin early to analyse the potential cost-effectiveness of trying to manage contractures. It provides data that suggests that the perceived expense of botulinum toxin early after a stroke before any contractures have developed may help reduce health costs longer term. Clinicians, often subconsciously, make decisions based on the cost of treatment on a daily basis. This is a form of rationing services which clinicians are often unaware they make. It is uncomfortable for clinicians who want to be patient-centred but must balance this ideal, with remaining service-centred to ensure patient flow through their service. We hope that this study helps to ease just one of the many cost-based treatment decisions that clinicians must make.

Treating spasticity early, in stroke patients at risk of contractures, with botulinum toxin does not lead to a significant increase in cost associated with managing these patients. Future-powered studies should now focus on the long-term cost-effectiveness associated with treatment involving botulinum toxin and ensure all social costs are included to allow for a more meaningful result.

**Clinical messages**

- The early use of botulinum toxin, as soon as spasticity is identified, appears to be cost-neutral.
- Mean contracture costs for the treatment group were £817 while the costs for the control group were £2298 (*p* = 0.04).
- Both base case results for cost per improvement of Barthel and Action Research Arm Test scores indicate that the intervention costs less and is more effective.

## References

[1] Patel A Berdunov V Quayyum Z, et al. Estimated societal costs of stroke in the UK based on a discrete event simulation. Age Ageing 2020; 49: 270–276. 31846500 10.1093/ageing/afz162 PMC7047817

[2] Lundstrom E Smits A Borg J, et al. Four-fold increase in direct costs of stroke survivors with spasticity compared with stroke survivors without spasticity: The first year after the event. Stroke 2010; 41: 319–324. 20044535 10.1161/STROKEAHA.109.558619

[3] Raluy-Callado M Cox A MacLachlan S, et al. A retrospective study to assess resource utilization and costs in patients with post-stroke spasticity in the United Kingdom. Curr Med Res Opin 2018; 34: 1317–1324. 29490512 10.1080/03007995.2018.1447449

[4] Cousins E Ward A Roffe C, et al. Does low-dose botulinum toxin help the recovery of arm function when given early after stroke? A phase II randomized controlled pilot study to estimate effect size. Clin Rehabil 2010; 24: 501–513. 20483887 10.1177/0269215509358945

[5] Lindsay C Ispoglou S Helliwell B, et al. Can the early use of botulinum toxin in post stroke spasticity reduce contracture development? A randomised controlled trial. Clin Rehabil 2021; 35: 399–409. 33040610 10.1177/0269215520963855 PMC7944432

[6] Shackley P Shaw L Price C, et al. Cost-effectiveness of treating upper limb spasticity due to stroke with botulinum toxin type A: Results from the botulinum toxin for the upper limb after stroke (BoTULS) trial. Toxins (Basel) 2012; 4: 1415–1426. 23342679 10.3390/toxins4121415 PMC3528253

[7] Makino K Tilden D Guarnieri C, et al. Cost effectiveness of long-term incobotulinumtoxin-A treatment in the management of post-stroke spasticity of the upper limb from the Australian payer perspective. PharmacoEconomics-open 2019; 3: 93–102. 29915932 10.1007/s41669-018-0086-z PMC6393278

[8] Lazzaro C Baricich A Picelli A, et al. Abobotulinumtoxina and rehabilitation vs rehabilitation alone in post-stroke spasticity: A cost-utility analysis. J Rehabil Med 2020; 52: 1–9. 10.2340/16501977-2636 31820010

[9] Lindsay C Simpson J Ispoglou S, et al. The early use of botulinum toxin in post-stroke spasticity: study protocol for a randomised controlled trial. Trials 2014; 15: 1–5. 24401159 10.1186/1745-6215-15-12 PMC3895790

[10] British Medical Association. Royal Pharmaceutical Society of Great Britain. In: British national formulary: BNF. 77 March 2019–September 2019. London, BMA: Royal Pharmaceutical Company, 2019.

[11] Radensky PW Archer JW Dournaux SF, et al. The estimated cost of managing focal spasticity: a physician practice patterns survey. Neurorehabil Neural Repair 2001; 15: 57–68. 11527280 10.1177/154596830101500108

[12] Fleuren JF Voerman GE Erren-Wolters CV, et al. Stop using the Ashworth scale for the assessment of spasticity. J Neurol Neurosurg Psychiatry 2010; 81: 46–52. 19770162 10.1136/jnnp.2009.177071

[13] Pandyan AD Vuadens P van Wijck FM, et al. Are we underestimating the clinical efficacy of botulinum toxin (type A)? Quantifying changes in spasticity, strength, and upper limb function after injections of Botox to the elbow flexors in a unilateral stroke population. Clin Rehabil 2002; 16: 654–660. 12392341 10.1191/0269215502cr536oa

